# Integrative transcriptomics and metabolomics reveal Cry1-associated hepatic alterations and plasma essential fatty acids as biomarkers of residual circadian disruption post-sepsis

**DOI:** 10.3389/fendo.2026.1916119

**Published:** 2026-10-01

**Authors:** Junzhou Zhao, Tianxiao Huang, Li Wang, Yun Shi, Wanni Zou, Yunxuan Chen, Qisheng Su, Linlan Qiao, Zheng Wu, Yi Lv, Yuanyuan Zhang, Rongqian Wu

**Affiliations:** 1 Department of Hepatobiliary Surgery, The First Affiliated Hospital of Xi’an Jiaotong University, Xi’an, China; 2 National Local Joint Engineering Research Center for Precision Surgery and Regenerative Medicine, Shaanxi Provincial Center for Regenerative Medicine and Surgical Engineering, The First Affiliated Hospital of Xi’an Jiaotong University, Xi’an, China; 3 Department of Cell Biology and Physiology, The University of Kansas Medical Center, Kansas City, KS, United States

**Keywords:** alpha-linolenic, circadian rhythm dysfunction, *Cry1*, entrainment, linoleic acid, sepsis

## Abstract

**Background:**

Sepsis disrupts circadian rhythm, leaving lasting molecular traces and circadian insensitivity to jet lag even after acute phase recovery. However, the core mechanism and biomarkers of residual circadian dysfunction post-sepsis remain unclear, which hinders targeted interventions.

**Methods:**

Male mice were subjected to non-lethal LPS injection, and after 7 days of recovery, liver transcriptomics and plasma metabolomics were integrated. Behavioral, physiological, and molecular analyses were performed, with statistical tests including DESeq2, Student’s t-test, MetaCycle, and ROC curve analysis.

**Results:**

Significant transcriptomic alterations were observed, most prominently at Zeitgeber time 21. Linoleic acid (LA) and alpha-linolenic acid (ALA) were identified as sampling-time-independent candidate biomarkers associated with post-sepsis circadian disruption. Exogenous supplementation of these two fatty acids shortened male mice’s rhythm re-entrainment period.

**Conclusions:**

This study identifies a hypothesized *Cry1*-essential fatty acid axis potentially contributing to post-sepsis circadian dysfunction based on multi-omics associations, providing novel biomarkers and a metabolic intervention strategy for detecting and treating circadian rhythm disorders in sepsis survivors.

## 1 Introduction

Sepsis is a life-threatening organ dysfunction arising from a dysregulated host response to infection, and remains one of the leading causes of mortality in critical care medicine, responsible for an estimated 49 million incident cases and 11 million deaths annually worldwide, accounting for approximately 20% of all global mortality. Although some patients survive the acute phase, they frequently endure chronic sequelae, including long-term cognitive impairment, sleep disturbances, and persistent fatigue, which substantially diminish quality of life and contribute to elevated long-term mortality risk (1–3). Population-based cohort data demonstrated that 6.4 % of sepsis survivors develop newly-onset sleep disorders within one-year post-discharge, and sleep-circadian disturbances are independently associated with elevated 5-year all-cause mortality in this population (4). Among general ICU-hospitalized patients, circadian misalignment and sleep architecture disruption are highly prevalent, occurring in more than 60 % of critically-ill subjects, and these rhythm abnormalities are tightly linked to delirium, prolonged mechanical ventilation dependency and delayed clinical recovery (5). In the past few decades, research on sepsis has focused on acute cytokine storms, organ damage, and life support treatments, significantly improving patients’ acute survival rates (6, 7). However, there are still significant shortcomings in exploring chronic pathophysiological changes, molecular mechanisms, and intervention targets after sepsis recovery. Clinical cohort studies have confirmed that survivors of sepsis generally suffer from sleep-wake disorders, decreased ability to synchronize circadian rhythms, and jet lag syndrome, which are closely related to cognitive decline, depression and anxiety, and inflammation relapse (8–11). However, the core mechanism of circadian rhythm disorders after sepsis recovery has not been elucidated, and there is a lack of targeted intervention strategies, which has become a key bottleneck restricting the long-term recovery of sepsis patients.

The circadian rhythm is a 24-hour periodic physiological rhythm formed by organisms adapting to the Earth’s rotation. It is regulated by the central circadian clock of the hypothalamic suprachiasmatic nucleus (SCN) and the circadian clock of peripheral tissues. In recent years, the interaction between circadian rhythm and critical illness has become a research hotspot. Light dark disorder, mechanical ventilation, sedation and analgesia in the intensive care unit (ICU) environment can further exacerbate circadian rhythm disorders, which can worsen sepsis prognosis by amplifying the inflammatory response, damaging mitochondrial function, and disrupting metabolic stability (12–14). Research has found that acute sepsis can significantly inhibit the rhythmic expression of core clock genes such as *Clock*, *Bmal1*, *Per1/2*, and *Cry1/2*, etc., reduce the amplitude of rhythms, and lead to systemic rhythm disorders (15–17). As a prototypical peripheral circadian clock organ, the liver orchestrates a large fraction of rhythmic gene expression to coordinate systemic lipid, carbohydrate and inflammatory homeostasis, receiving hierarchical input from the hypothalamic suprachiasmatic nucleus while maintaining cell-autonomous clock oscillation (18). The liver exhibits high susceptibility to systemic inflammatory stress during sepsis (19). Critically, even after acute inflammatory mediators subside, hepatic tissues can retain long-term molecular memory of prior inflammatory injury, driving sustained transcriptomic and metabolic reprogramming (20). These properties render the liver an ideal tissue to probe residual circadian impairments post-sepsis and bridge core clock transcriptional dysregulation to circulating plasma metabolite alterations observed in sepsis survivors. After the recovery of sepsis, even if the acute phase inflammation subsides and organ function recovers, the synchronization ability of the biological clock continues to be impaired. The mechanism of injury is not simply residual inflammatory effects, but involves a complex process of long-term metabolic reprogramming and molecular rhythm remodeling. Currently, research on related mechanisms is still in a blank stage.

Sepsis, a systemic metabolic disorder, is accompanied by comprehensive metabolic reprogramming of lipids, glucose, amino acids, and other metabolic processes. Among them, polyunsaturated fatty acids (PUFAs) play a key role in rhythm regulation, inflammation regulation, and neural function maintenance as components of cell membrane structure and signaling molecules. Linoleic acid (LA, ω-6) and alpha linolenic acid (ALA, ω-3) are essential polyunsaturated fatty acids in the human body that cannot be synthesized on their own and require dietary intake. They not only are important maintenance factors for cell membrane fluidity, but also regulate gene transcription through pathways such as peroxisome proliferator activated receptors (PPARs) and G protein coupled receptors (GPR120), participating in inflammation and metabolic homeostasis regulation (21–25).The latest research has confirmed that polyunsaturated fatty acids are important non photosynthetic circadian synchronization signals, which can affect the phase and synchronization efficiency of the biological clock by regulating PER2 protein phosphorylation and the core clock gene transcription rhythm. An imbalance in the ratio of omega-3/omega-6 fatty acids can directly lead to rhythm disorders and a decreased ability to adapt to time differences (26, 27). At the same time, there are significant lipid metabolism disorders during the course of sepsis, with increased fat breakdown and abnormal fatty acid oxidation in the acute phase, and persistent deficiency of essential fatty acids in the recovery phase, which is associated with long-term neurocognitive dysfunction (28–30). The intergrated analysis of the transcriptome and metabolome, as a core tool for analyzing the molecular mechanisms of diseases, can systematically reveal the correlation between gene expression and metabolite changes. It has been widely used in the study of metabolic disorders and rhythm regulation in sepsis, providing a powerful tool for screening key regulatory molecules and pathways (31, 32).

At present, the metabolic mechanism underlying the weakened ability to synchronize circadian rhythms after sepsis recovery is not clear, and there is no experimental evidence to determine whether essential fatty acid deficiency is the core driving factor. Moreover, there is a lack of effective treatment methods to improve the long-term rhythm disorder of sepsis survivors. Based on the existing research gaps, we propose a hypothesis: Non-lethal LPS exposure induces persistent dysregulation of the core hepatic clock gene *Cry1*, which may in part reshape the rhythmic transcriptomic landscape of the liver and be statistically associated with sustained reduction of plasma linoleic acid and alpha-linolenic acid; these fatty-acid changes could ultimately impair circadian re-entrainment ability. We note that this causal regulatory relationship remains to be formally verified by further genetic experiments; furthermore, exogenous supplementation of these two essential fatty acids can reverse post-sepsis circadian dysfunction by restoring clock metabolic homeostasis. Specifically, we predict that post-LPS residual circadian dysfunction may not be a non-specific residual inflammatory outcome, but a specific molecular event dominated by core circadian genes mediated hepatic transcriptional alterations, which may further triggers stable plasma essential fatty acid metabolic disorders; these lipid metabolites may serve as reliable biomarkers for residual circadian disruption, and their deficiency is a core functional driver of impaired circadian synchronization in sepsis recovery.

Based on this, this study focused on the key scientific issue of circadian rhythm synchronization disorder after sepsis recovery. A sepsis survival mouse model was constructed via non-lethal LPS injection, and the time difference adaptation ability was evaluated through rotation activity rhythm detection. Combined with transcriptome and metabolome analysis, key differential metabolites were screened to clarify the significant reduction in linolenic acid and linoleic acid in sepsis recovered mice. The rescue effect of both on rhythm synchronization ability was verified through exogenous supplementation experiments. The aim of this study was to reveal a new mechanism by which essential fatty acid metabolism reprogramming mediates the dysfunction of the biological clock after sepsis, provide a novel metabolic intervention strategy for long-term rhythm disorders and neurological rehabilitation in sepsis survivors, and fill the research gap in the understanding of the mechanism of chronic rhythm damage and targeted interventions after sepsis.

## 2 Materials and methods

### 2.1 Animals and phase shift models

This study used 8-week-old C57BL/6 mice (SLAC animals). All animals were purchased from the Experimental Animal Center of Xi’an Jiaotong University (Xi’an, China) and brought into a rotating cage under 12 hours of light/dark conditions. To generate *Cry1* and *Cry2* double-knockout (DKO) mice, mice carrying individual null alleles of *Cry1* and *Cry2* were interbred, and homozygous DKO mice were obtained through mating and PCR-based genotyping. All the mice were allowed free access to obtain food and water. The beginning of light is defined as Zeitgeber time 0 (ZT0), and the beginning of darkness is defined as ZT12. When the light on the running wheel lights up, the light intensity at the bottom of the cage is about 100 lux. The experimental group of mice were given a non-lethal concentration of LPS 5 mg/kg, while the control group mice were given the same dose of physiological saline, and the dark cycle was advanced by 6 hours after 7 days (The instantaneous rhythm disruption caused by acute stimulation often takes about 7 days to recover (32, 33). The entire process involved placing the mice in the comprehensive lab animal monitoring system (PhenoMaster, TSE, Germany).All experiments were conducted using male mice, except were indicated in **Supplementary Figure S6**.

### 2.2 Sample collection

Collect liver and plasma samples every 4 hours within 24 hours, starting from ZT16 on the 6th day and collecting ZT12 samples every 4 hours until the 7th day. All animals were sampled within 30 min before each Zeitgeber time point; thus, tissues at ZT0 and ZT12 were collected before lights-on or lights-off, avoiding acute light-induced effects. Liver samples were rapidly frozen, ground into a fine powder in liquid nitrogen, and stored at −80 °C for further analysis. Blood samples were drawn from the inferior vena cava, collected in heparin-coated anticoagulant tubes, centrifuged to obtain the plasma, and promptly stored at −80 °C. Liver samples and plasma samples were obtained from the same mice and subsequently used for RNA-Seq and untargeted metabolomics, respectively.

### 2.3 Running wheel activity

To detect locomotor activity, the mice were individually housed in a running wheel-equipped cage, and wheel rotation was recorded throughout the experiments. Entrainment was defined as stable activity onset or offset, whichever occurred later, for 4 consecutive days or days before sample collection if fewer than 4.

### 2.4 Enzyme-linked immunosorbent assay

ELISA kits were used to measure the plasma corticosterone (Mlbio, ml058380),melatonin contents (Mlbio, ml002209), TNFα (Mlbio, ml002095) and IL-6 (Mlbio, ml063159) as directed by the manufacturer, following standard procedures.

### 2.5 Aspartate aminotransferase and alanine aminotransferase measurement

The mouse AST assay kit (C010-2, Nanjing Jiancheng Bioengineering Institute, Nanjing, China) and ALT assay kit (C009-2, Nanjing Jiancheng Bioengineering Institute, Nanjing, China) were used for the detection of AST and ALT according to the manufacturer’s instructions.

### 2.6 Quantitative real-time PCR

RNA extraction, reverse transcription, and quantitative real-time PCR were conducted as we previously described (34). β-actin was used as an endogenous control. The primers (TAKARA BIO INC, China) used in this study are shown in **Supplementary Table S1**.

### 2.7 RNA-Seq

Extract total RNA using TRIzol reagent. The RNA was quantified and identified via an RNA Nano 6000 assay kit on the Bioanalyzer 2100 system (Agilent Technologies, California, USA) to assess RNA integrity. Total RNA extracted from liver tissue was used as input material for RNA sample preparation in transcriptome sequencing libraries. RNA sequencing, mapping, and gene/transcript quantification were performed by Genergy (Shanghai, China). Select cDNA fragments of 370–420 bp were selected, and library fragments were purified via using the AMPure XP system (Beckman Coulter, Beverly, USA) with a length of bp. Then use Phusion high fidelity DNA polymerase, universal PCR primers, and Index (X) primers for PCR. Finally, the PCR products were purified using the AMPure XP system and the library quality was evaluated via the Agilent Bioanalyzer 2100 system.

### 2.8 Untargeted metabolomics

Metabolic analysis was performed using the ACQUITY UPLC I-Class system (Waters Corporation, Milford, USA) and VION IMS QTOF mass spectrometer (Waters Corporation) in ESI positive and negative ion modes by Shanghai Bioprofile (Shanghai, China). Chromatographic separation was achieved on an ACQUITY UPLC BEH C18 column (1.7 μm, 2.1 × 100 mm) maintained at 40 °C. The mobile phase consisted of (A) 0.1% formic acid in water and (B) 0.1% formic acid in acetonitrile, with a gradient elution as follows: 0–2 min, 2% B; 2–15 min, 2%–98% B; 15–17 min, 98% B; 17–17.5 min, 98-2% B; 17.5–20 min, 2% B. The flow rate was 0.3 mL/min, and the injection volume was 2 μL.

Mass spectrometry data were acquired with collision energies of 10, 20, and 40 eV. Raw LC–MS data were processed for peak detection, alignment, and integration. Metabolite annotation was performed by matching experimental data against public databases, including the Human Metabolome Database (HMDB; http://www.hmdb.ca), LIPID MAPS (http://www.lipidmaps.org), MassBank, and the Global Natural Products Social Molecular Networking (GNPS) database, as well as an in-house Bioprofile metabolite database (BP-DB). Annotation confidence was classified into four levels: Level 1, concordant precursor ion mass, tandem mass spectrometry (MS/MS) spectra, and retention time (RT); Level 2, concordant precursor ion mass and MS/MS spectra; Level 3, concordant precursor ion mass and RT; and Level 4, concordant precursor ion mass only. Only metabolites annotated at Level 1 or Level 2 were included in subsequent analyses. Raw metabolite intensities were normalized to the internal standard and subsequently scaled to the total ion current (TIC), with the final values expressed as relative intensities.

All the chemicals and solvents used were of analytical grade or HPLC grade. Water, methanol, acetonitrile, and formic acid were purchased from CNW Technologies GmbH (Dusseldorf, Germany). The internal standard L-2-chloroalanine was sourced from Shanghai Bioprofile Co., Ltd.

### 2.9 Indirect calorimetry and food intake measurement

Whole body metabolic status was tested using indirect calorimetry in the comprehensive lab animal monitoring system (PhenoMaster, TSE, Germany). The metabolic chamber is used to measure various metabolic parameters, including oxygen consumption, carbon dioxide production, and food intake. After adapting to the environment for one week, continuously monitor and record the changes in these parameters of mice for 14 days or longer. During this period, the system automatically collects parameter data every 2 minutes.

### 2.10 Feeding with linoleic acid and alpha- linolenic acid

Feeding a diet containing linoleic acid and alpha-linolenic acid in a 1:1 ratio (4% each, with a total fat content of 15% E) (35, 36) in LA+ALA group; the control group was fed with normal feed and the isocaloric matched-control group (LA and ALA < 0.5% E, with a total fat content of 15% E). All the animals were allowed free access to food during the experiment. Throughout the entire experimental process, diurnal exercise activity and basal metabolic parameters (such as food intake and activity counts) were monitored to evaluate the regulatory effects of feeding with linoleic acid and alpha linolenic-linolenic acid on circadian rhythms.

### 2.11 Statistics

An integrative analysis of transcriptomic and metabolomic data was performed. Transcriptomic data were analyzed based on raw count matrices using the DESeq2 (v1.48.2). Differentially expressed genes (DEGs) were identified using a threshold of adjusted p-value (p.adj) < 0.05 and |log2 fold change| > 1. For metabolomic data, differential analysis was conducted using Student’s t-test, followed by false discovery rate (FDR) correction. Differential metabolites were defined as those with FDR-adjusted p-values < 0.05.

Circadian rhythm analyses were performed using the MetaCycle (v1.2.1) and dryR (v1.0.0). Rhythmicity was defined as p < 0.05. Genes were classified as rhythmic or non-rhythmic based on the JTK-Cycle algorithm implemented in MetaCycle. Furthermore, dryR was used to classify genes into five models, representing distinct patterns of rhythmic changes between the two groups.

GO and KEGG pathway enrichment analysis were conducted using the WEI SHENG XIN online platform (https://www.bioinformatics.com.cn/). Pathways with p < 0.05 were considered significantly enriched.

Orthogonal partial least squares discriminant analysis (OPLS-DA) was performed using the ropls (v1.40.0). These analyses were used to assess global sample distribution and group separation. Receiver operating characteristic (ROC) curve analysis was performed to evaluate the discriminative performance of candidate biomarkers using the pROC (v1.19.0.1). The area under the curve (AUC) was calculated to quantify diagnostic accuracy.

To investigate transcription factor binding patterns, motif enrichment analysis was performed on selected genomic regions. *De novo* motif discovery was conducted using the MEME Suite (v5.5.7). For targeted analysis of *Cry1*, motif scanning and enrichment were performed using HOMER with ChIP-seq data from ChIP-Atlas (hg38). Motif profiles of other circadian transcription factors were obtained from the JASPAR database.

All statistical analyses were performed using GraphPad Prism (v10.4.1) and R (v4.5.0). Comparisons between two groups were conducted using two-tailed Student’s t-tests. For multiple group comparisons, one-way or two-way analysis of variance (ANOVA) followed by Tukey’s *post hoc* test was applied. A p-value < 0.05 was considered statistically significant. Data visualization was performed using ggplot2 (v4.0.2). Heatmaps were generated using ComplexHeatmap (v2.26.1) and pheatmap (v1.0.13).

## 3 Results

### 3.1 Behavioral and physiological changes in mice on day 7 after non-lethal does LPS injection

In order to detect the relevant physiological conditions of mice after sepsis recovery, we injected non-lethal doses of LPS (5 mg/kg) into the mice (**Supplementary Figure S1A**). After 7 days of LPS injection, there was no significant difference in the liver weight ratio or the spleen weight ratio between the LPS group and the normal control (NC) group (**Supplementary Figure S1B**). The melatonin levels in the LPS group also returned to normal levels, reaching their peak at ZT13 and their trough at ZT1. The liver function of the LPS group also returned to normal levels, but the level of glucocorticoids in the LPS group was significantly lower than those in the NC group at all time points, which may be due to the depletion of corticosteroids after the acute phase (**Supplementary Figure S1C–E**). Moreover, the plasma TNFα and IL-6 levels in the LPS group also returned to normal levels (**Supplementary Figure S1F**). In addition, there was no significant difference in metabolic related indicators and food intake between the two groups (**Supplementary Figure S1G–I**). However, compared to NC group, mice exposed to LPS increased the time of re entrainment, although activity did not significantly decrease(**Supplementary Figure S2**).

### 3.2 Whole transcriptomics analysis of the liver

To reveal the changes in liver gene expression in the mice after sepsis recovery, we collected liver samples every four hours on the seventh day after LPS injection for RNA-seq. We first analyzed all the time points together. PCA of transcriptomic profiles across all sampled ZT time points showed substantial overlap between LPS and NC samples, potentially due to confounding effects from time-related variation, with PC1 and PC2 explaining 24.49% of the total variance (**Supplementary Figure S3A**). Orthogonal partial least squares discriminant analysis(OPLS-DA) showed that the two groups were clearly separated (**Supplementary Figure S3B**). The volcano plot displays the upregulated and downregulated genes between the two groups, with those showing a > fourfold change individually marked (**Figure 1A**).

**Figure 1:**
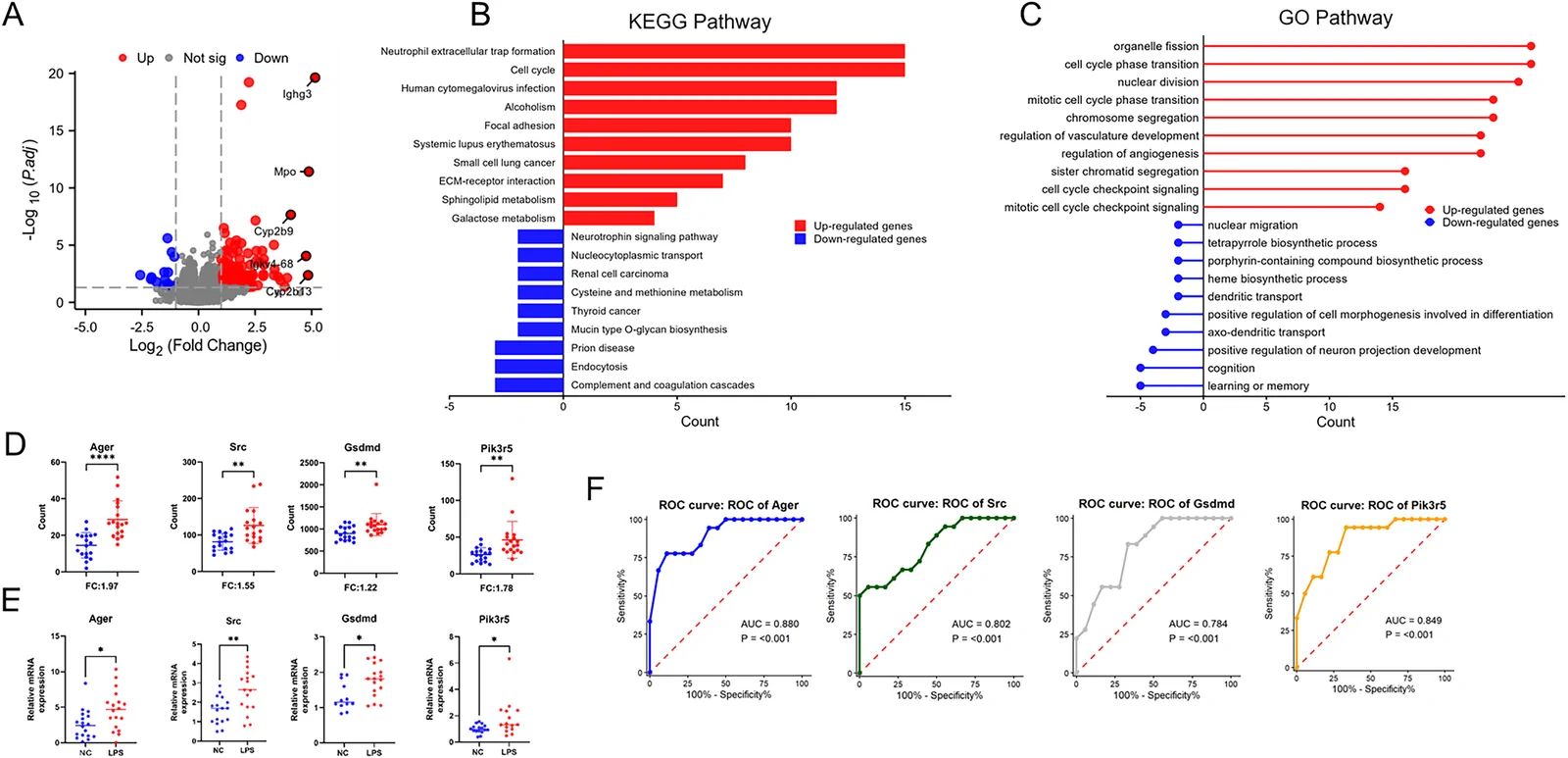
Whole-transcriptome analysis of liver samples combined across six circadian time points within a 24-hour cycle. **(A)** Volcano plot of differentially expressed genes (DEGs) between the LPS group and NC group. **(B)** KEGG pathway enrichment of upregulated and downregulated genes. **(C)** GO analysis of upregulated and downregulated genes. **(D)** Immune-related DEGs identified from transcriptome analysis (n = 18). **(E)** qPCR validation of immune-related DEGs (n = 18). **(F)** ROC curve analysis of immune-related downregulated genes (n = 18). *p < 0.05; **p < 0.01; ****p < 0.0001.

The enrichment analysis of differentially expressed genes (DEGs) by GO and KEGG showed that several inflammation related pathways and cell proliferation related pathways were upregulated in the LPS group, including neutrophil extracellular trap formation, cell cycle, organelle fission and nuclear division. In contrast, the downregulated genes were mainly enriched in pathways related to complement and coagulation cascades, and heme biosynthetic process. These results reflect sustained chronic inflammation in mice after LPS stimulation, accompanied by compensatory proliferation and functional inhibition of liver cells (**Figures 1B,C**).

The top 10 DEGs are presented in the table (**Supplementary Figure S3C**). We validated the highest ranked immune related genes in an independent batch of liver tissues through qPCR, including *Ager*, *Src*, Gsdmd and *Pik3r5*. ROC curve analysis confirmed significant differences between LPS and NC groups (**Figures 1D–F**).

### 3.3 Circadian transcriptomics analysis of the liver

We analyzed the rhythmicity of the transcriptome through JTK_cycle analysis. In the NC group, 4417 genes were identified as rhythmic, while 1919 rhythmic genes were found in the LPS group, with 1053 rhythmic genes overlapping between the two groups (**Figure 2A**). To further analyze the rhythm patterns, we applied dryR, which divides genes into five different models.

**Figure 2:**
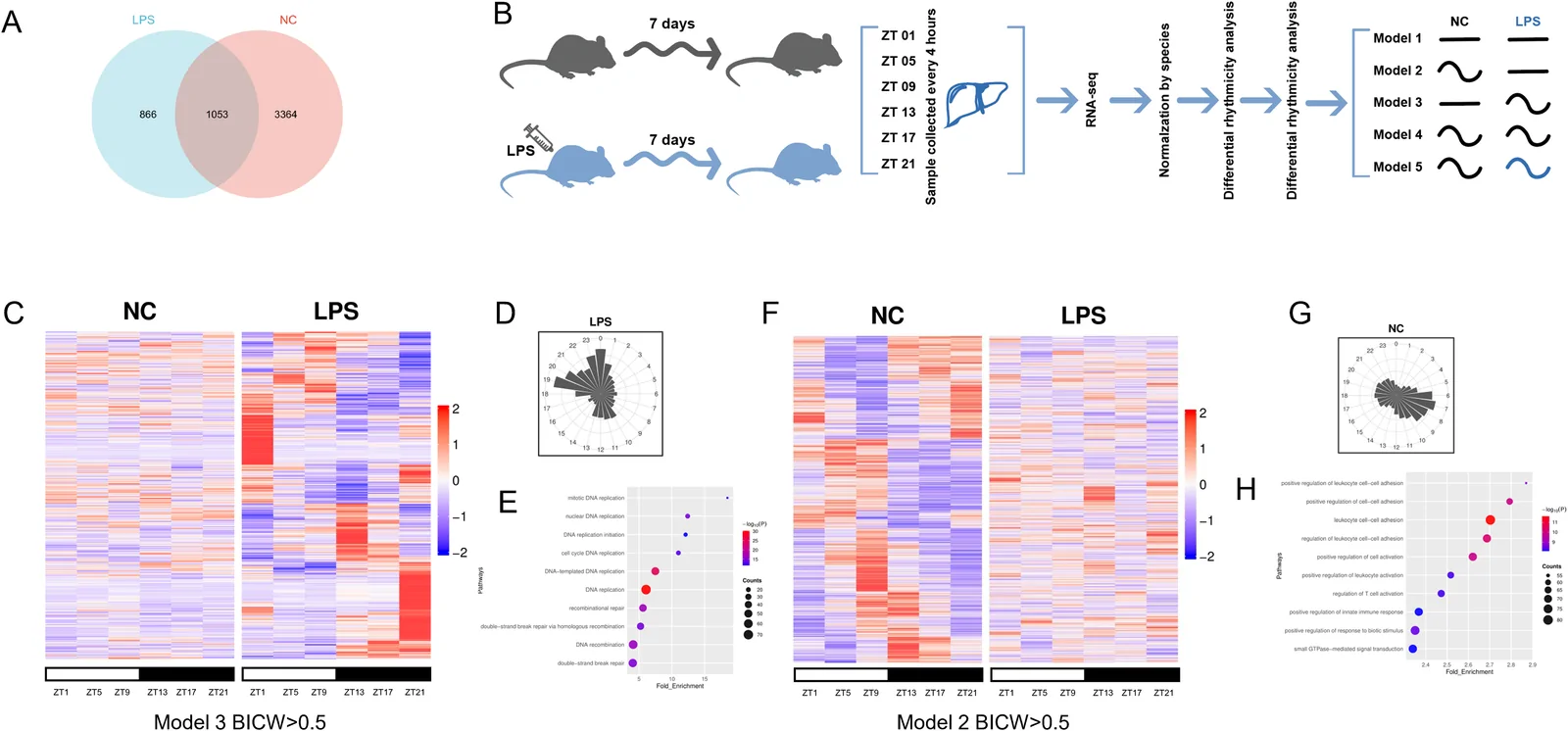
Analysis of circadian rhythmicity in liver transcriptomes across a 24-hour cycle. **(A)** Venn diagram of rhythmic genes in NC and LPS groups. **(B)** Classification of transcriptome profiles using dryR. **(C)** Heatmap of genes in Model 3 from dryR classification. **(D)** Circadian phase distribution visualized by circular plot. **(E)** KEGG enrichment of Model 3 genes (n = 3). **(F)** Heatmap of genes in Model 2 from dryR classification. **(G)** Circular plot of circadian phase distribution. **(H)** KEGG enrichment of Model 2 genes (n = 3).

Models 1 (JTK-Cycle p > 0.05 in NC group, p < 0.05 in LPS group; sepsis recovery triggers novel 24-h periodic transcription that is absent under physiological conditions) and Models 4 (JTK-Cycle p < 0.05 in both groups; no statistically significant shift in peak/trough phase and no difference in oscillation amplitude between groups) represent the largest set of genes in the two groups that lack rhythmicity or exhibit similar rhythms (**Supplementary Figure S4A–F**). Model 2 represents genes with rhythm only in the control group (JTK-Cycle p < 0.05 in NC group, p > 0.05 in LPS group; baseline oscillatory pattern fully ablated post-LPS administration). Model 3 represents that only the LPS group has rhythm (JTK-Cycle p > 0.05 in NC group, p < 0.05 in LPS group; recovery after LPS administration triggers novel 24-h periodic transcription that is absent under physiological conditions) (**Figure 2B**).

Model 5 indicated that rhythmic patterns or modifys the amplitude or period of existing rhythms have changed in the LPS group (JTK-Cycle p < 0.05 in both groups; no statistically significant shift in peak/trough phase and no difference in oscillation amplitude between groups), with enrichment in cholesterol biosynthetic process via desmosterol, cholesterol biosynthetic process via lathosterol, and alpha amino acid metabolic process pathways (**Supplementary Figure S4G–I**).

Model 3 enriched in mitotic DNA replication, nuclear DNA replication, and DNA replication initiation pathways, which further indicated that regeneration related pathways were activated after LPS stimulation (**Figures 2C–E**).

Model 2 enriched in positive regulation of leukocyte cell-cell adhesion, positive regulation of cell-cell adhesion, and regulation of T cell activation, which suggests that the stability of the organizational structure may have been disrupted (**Figures 2F–H**).

Based on the transcriptome analysis above, we found that LPS reshaped the rhythmic changes at the transcriptome level of mouse liver. The LPS group had significantly fewer rhythmic genes than the NC group, indicating that the homeostasis of circadian rhythm genes was disrupted. These findings also demonstrate the potential long-term adverse effects of LPS on circadian rhythm disorders. Meanwhile, these changes also highlight the complexity of circadian rhythm regulation in metabolism and disease.

### 3.4 Expression patterns of core circadian clock genes in the liver

We further analyzed the expression patterns of classical core clock genes. Although the rhythmicity of these genes remains largely unchanged, significant transcriptional differences were observed at specific time points. For example, *Bmal1*, *Clock*, *Per1*, and *Cry1* show significant differences at ZT21, and there is a noticeable change in the peak phases of *Per1* and *Nr1d1* (**Figure 3A**). qPCR of independent liver samples confirmed the differences in gene expression levels and changes in the peak phase (**Figure 3B**). ROC curve analysis further confirmed significant differences in the expression of *Bmal1*, *Clock*, *Cry1*, and *Per1* between the two groups at ZT21 (**Figure 3C**). In addition, the heatmap visualization of the gene with the highest expression of ZT21 in the control group of mice revealed a significant loss of rhythmicity in the LPS group (**Figure 3D**).

**Figure 3:**
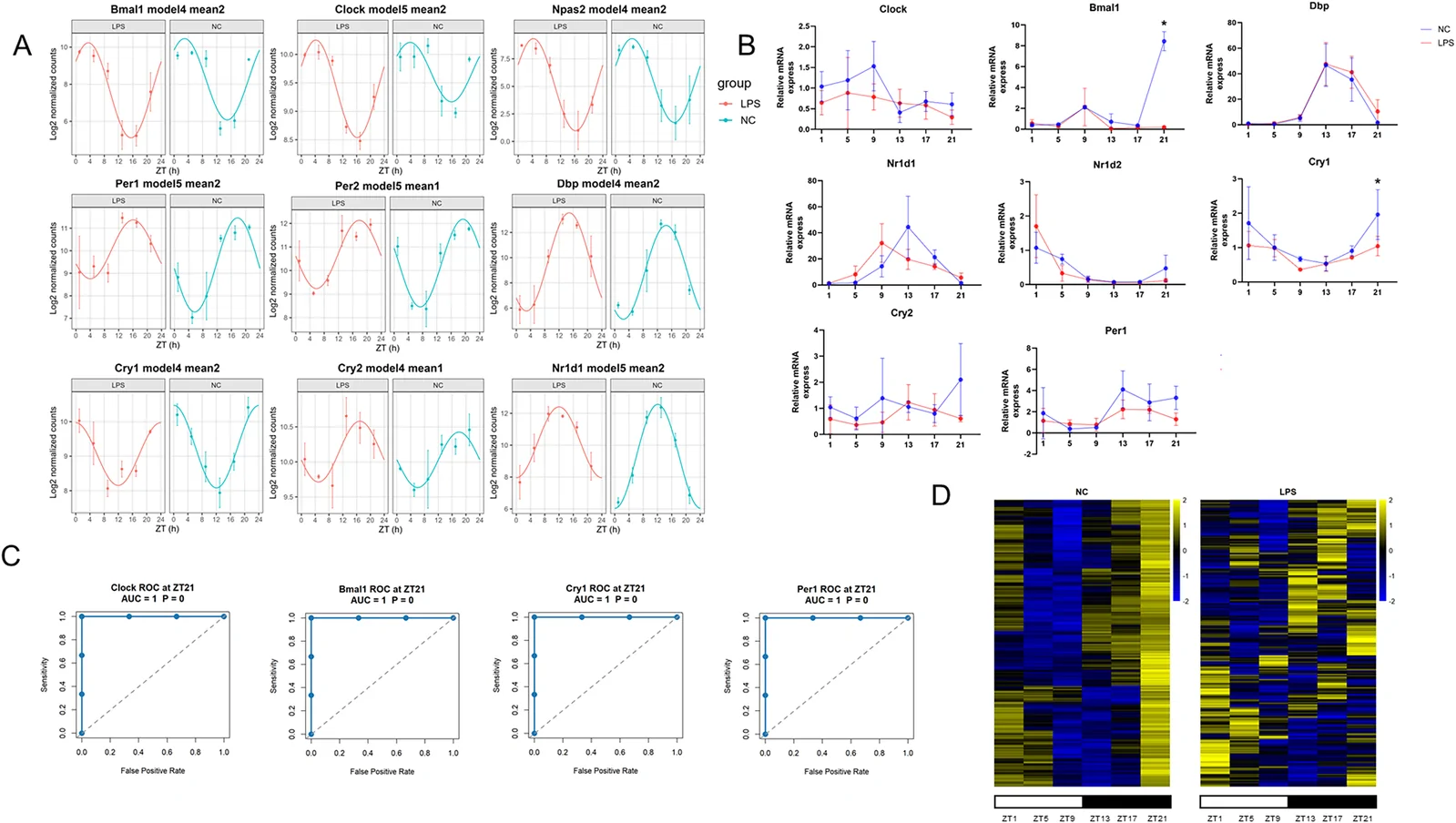
Expression patterns of core circadian clock genes in the liver transcriptomes across a 24-hour cycle. **(A)** Classification of core clock genes showing differences expression patterns based on dryR. **(B)** qPCR analysis of core clock genes 7 days after LPS injection (n = 3). **(C)** ROC curve analysis of Bmal1, Clock, Cry1, and Nr1d1 at ZT21. **(D)** Heatmap of core genes with peak expression at ZT21 in NC and LPS groups. *p < 0.05.

These findings indicate that after 7 days of non-lethal LPS injection, the expression of core clock genes was not fully restored, especially at ZT21. We speculate that changes in ZT21 gene expression, particularly in core genes including *Bmal1, Clock, Cry1*, and *Per1*, may be sensitive indicators of acute circadian rhythm disorders.

### 3.5 The reversibility of changes in core clock genes

To examine whether the changes in core clock gene expression are reversible and whether day 7 represents the recovery threshold. Based on the above results, we collected liver samples at ZT21 on days 0, 3, 5, 7, 9, and 11 after LPS injection (**Figure 4A**). As shown in **Figure 4B**, qPCR revealed that the expression of *Clock*, *Per1*, *Nr1d2*, and *Dbp* peaked on the 7th or 9th day and then returned to normal levels after LPS injection, while *Bmal1*, *Cry1*, *Cry2*, and *Nr1d1* did not return to normal levels during the 11 day observation period after LPS injection. These data suggest that *Bmal1*, *Cry1*, *Cry2*, and *Nr1d1* may serve as sensitive indicators of acute circadian rhythm dysregulation in mice that have recovered from LPS injection.

**Figure 4:**
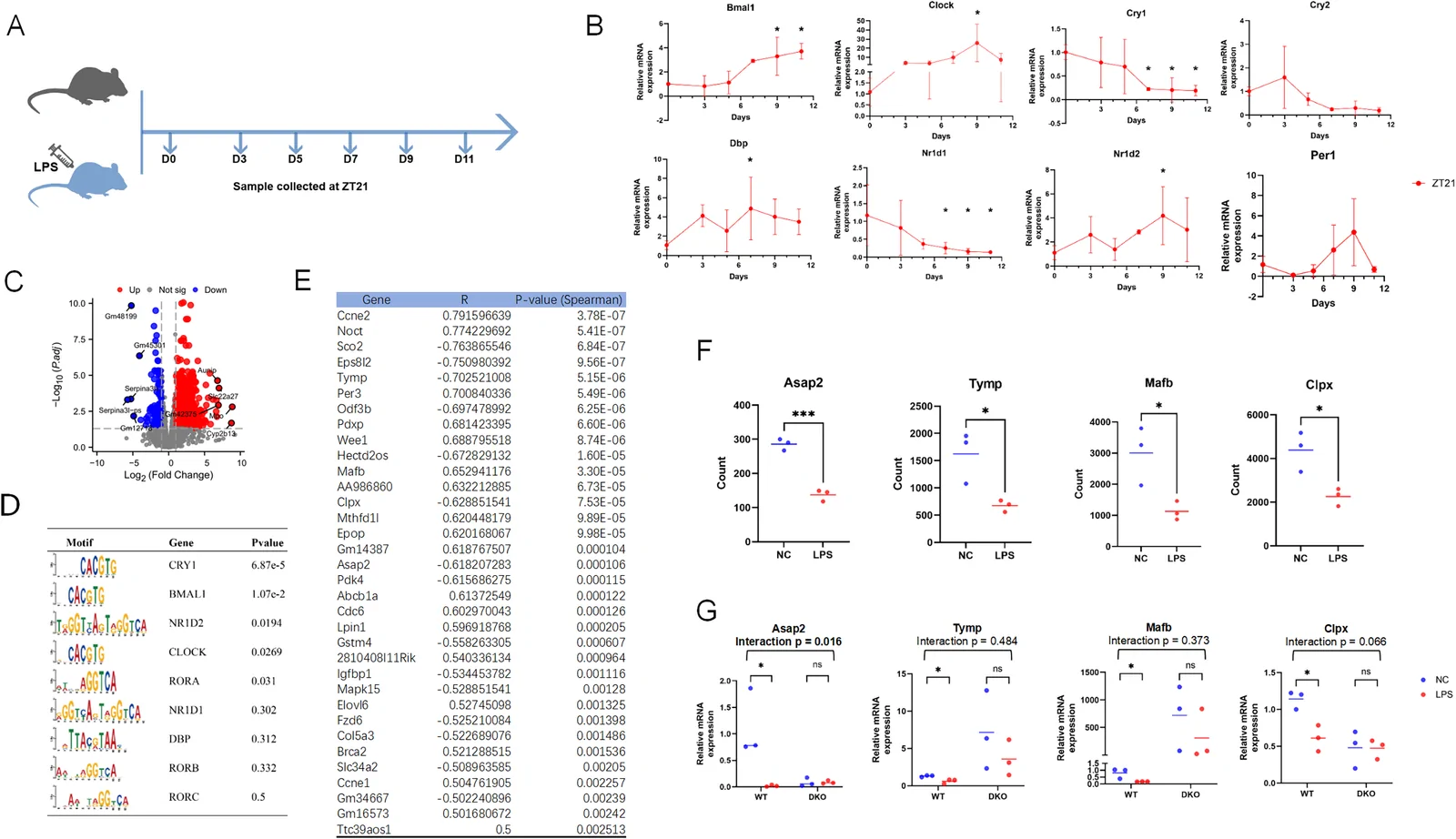
Transcriptomic analysis of liver at ZT21. **(A)** Schematic diagram of experiment. **(B)** Time-course qPCR analysis of core clock genes at ZT21 after LPS injection (n = 3). **(C)** Volcano plot of DEGs at ZT21 between NC and LPS groups. **(D)** Motif analysis of DEGs. **(E)** Top 22 DEGs most correlated with Cry 1. **(F)** mRNA level of Clpx, Mafb, Asap2 and Tymp (n =3). **(G)** Clpx, Mafb, Asap2 and Tymp expression in Cry1/Cry2 double knockout (DKO) mice and WT mice (n = 3), and their genotype-treatment interaction analysis between (WT: NC-LPS) and (DKO: NC-LPS). *p < 0.05; ***p < 0.001; ns, p>0.05.

### 3.6 Transcriptomic analysis of the liver at ZT21

Next, we focused on studying the transcriptome profile of ZT21 on the 7th day after LPS injection. A total of 689 DEGs were identified between the phase-shifted and control groups, with genes exceeding a fourfold change (|log_2_FC | > 2, adjusted p < 0.05) (**Figure 4C**). Motif analysis of these DEGs revealed that among the numerous core clock genes, *Cry1* is the most significant upstream core regulatory factor (**Figure 4D**). The correlation analysis between DEGs and *Cry1* identified 34 genes with strong correlation (|R|>0,5), which are mainly related to processes such as the cell cycle (*Ccne2*, *Cdc6*, *Asap2*, *Wee1*), circadian rhythm (*Per3*, *Noct*), energy metabolism (*Sco2*, *Clpx*, *Pdk4*), and structure and development (*Mafb*, *Odf3b*, *Col5a3*) (**Figures 4E, F**). We validated one representative DEG in each category using qPCR: *Clpx*, *Mafb*, *Asap2* and *Tymp*, confirming the results of transcriptome sequencing. To avoid the redundant effect of *Cry2*, we further constructed *Cry1* and *Cry2* double-knockout (DKO) mice. Of note, substantial intrinsic baseline differences in target-gene expression existed between untreated (WT-NC) and (DKO-NC) mice. These baseline shifts varied in both magnitude and direction across the four candidate genes, and could not be uniformly attributed to a simple repressor-function model of CRY proteins. Regardless of whether baseline expression was higher or lower in DKO relative to WT, these genotype-dependent constitutive differences represent a critical confounding factor when interpreting two-way ANOVA genotype-treatment interaction statistics. In DKO mice, LPS treatment failed to drive robust expression changes for these target genes. However, two-way ANOVA revealed a significant genotype-treatment interaction for Asap2 (p =0.016) and a similar, although non-significant, trend for Clpx (p = 0.066). In contrast, no significant genotype-treatment interactions were detected for Mafb or Tymp (p = 0.373 and 0.484, respectively). The genotype-treatment interaction effects of these genes are not significant, which may be due to significant individual sample differences (due to limited sample size) and the possible existence of non *Cry1* dependent pathways (**Figure 4G**). Although motif enrichment strongly predicted *Cry1* as a top upstream regulator of ZT21 DEGs, our DKO genetic experiment only provided partial experimental support for this regulatory model.

### 3.7 Behavioral, physiological, and gene expression changes in female mice

Multiple studies have shown that females are generally more adaptable to changes in time (37), and we evaluated whether female mice experienced similar changes. Seven days after LPS injection, there were no significant differences in behavioral activity or metabolic indicators compared to the NC group (**Supplementary Figure S6A–E**). However, at the mRNA level, qPCR analysis of liver samples showed significant downregulation of *Bmal1*, *Per1* and *Cry1* at ZT21, which was consistent with findings in males (**Supplementary Figure S6F**). These results suggest that although female mice may exhibit better behavioral adaptations to acute circadian rhythm dysregulation, they also demonstrate incomplete molecular recovery, particularly in the core clock gene of ZT21 seven days after LPS injection.

### 3.8 Plasma metabolomic analysis

Furthermore, to investigate potential effector molecules, we used LC/MS to explore potential plasma metabolite biomarkers in mice 7 days after LPS injection. PCA of metabolomic profiles across all sampled ZT time points showed substantial overlap between LPS and NC samples, potentially due to confounding effects from time-related variation, with PC1 and PC2 explaining 42.43% of the total variance (**Supplementary Figure S7A**). OPLS-DA showed a clear separation between the NC and LPS groups, suggesting distinct overall metabolic profiles between the NC group and the LPS group (**Figure 5A**). At all time points, the volcano plot identified several significantly altered metabolites (fold change ≥ 4): LysoPC(0:0/18:1(9Z)), Vinylamycin, and LysoPC (20:3 (8Z, 11Z, 14Z)/0:0) were significantly upregulated in the LPS group, LysoPC(17:0/0:0), 2-Methoxy-6-prenylhydroquinone and Thiazolidine-4-carboxylic acid were significantly decreased (**Supplementary Figure S7B, C**). ROC curve analysis confirmed that these three metabolites, either individually or in combination, exhibited strong discriminative power between the two groups (**Figure 5B**). To further investigate the changes at specific times, we clustered the significantly different metabolites at each time point in the heatmap (**Supplementary Figure S7D**), and listed the top 10 metabolites in the table (**Figure 5C**). In addition, the rhythmicity of some metabolites in the table, including 2-tetradecanoyl-sn-glycero-3-phosphocholine, 3-acetyl and LysoPC(0:0/18:1(9Z)), has also been altered (**Figure 5D**). These differential metabolites are divided into nine categories, among which lipids and lipid molecules are the most abundant (**Supplementary Figure S7E**). KEGG pathway analysis showed that these metabolites were mainly enriched in pathways such as Linoleic acid metabolism, Alcoholisms, Furfural degradation, etc. (**Figure 5E**). It is worth noting that Linoleic acid or alpha- Linolenic acid involved in Linoleic acid metabolism consistently decrease in the LPS group (**Figure 5F**). Other key metabolites related to Linoleic acid metabolism, including Crepenynic acid, alpha-Dimorphecolic acid and Stearidonic acid, also consistently decrease in the LPS group (**Supplementary Figure S7F**).

**Figure 5:**
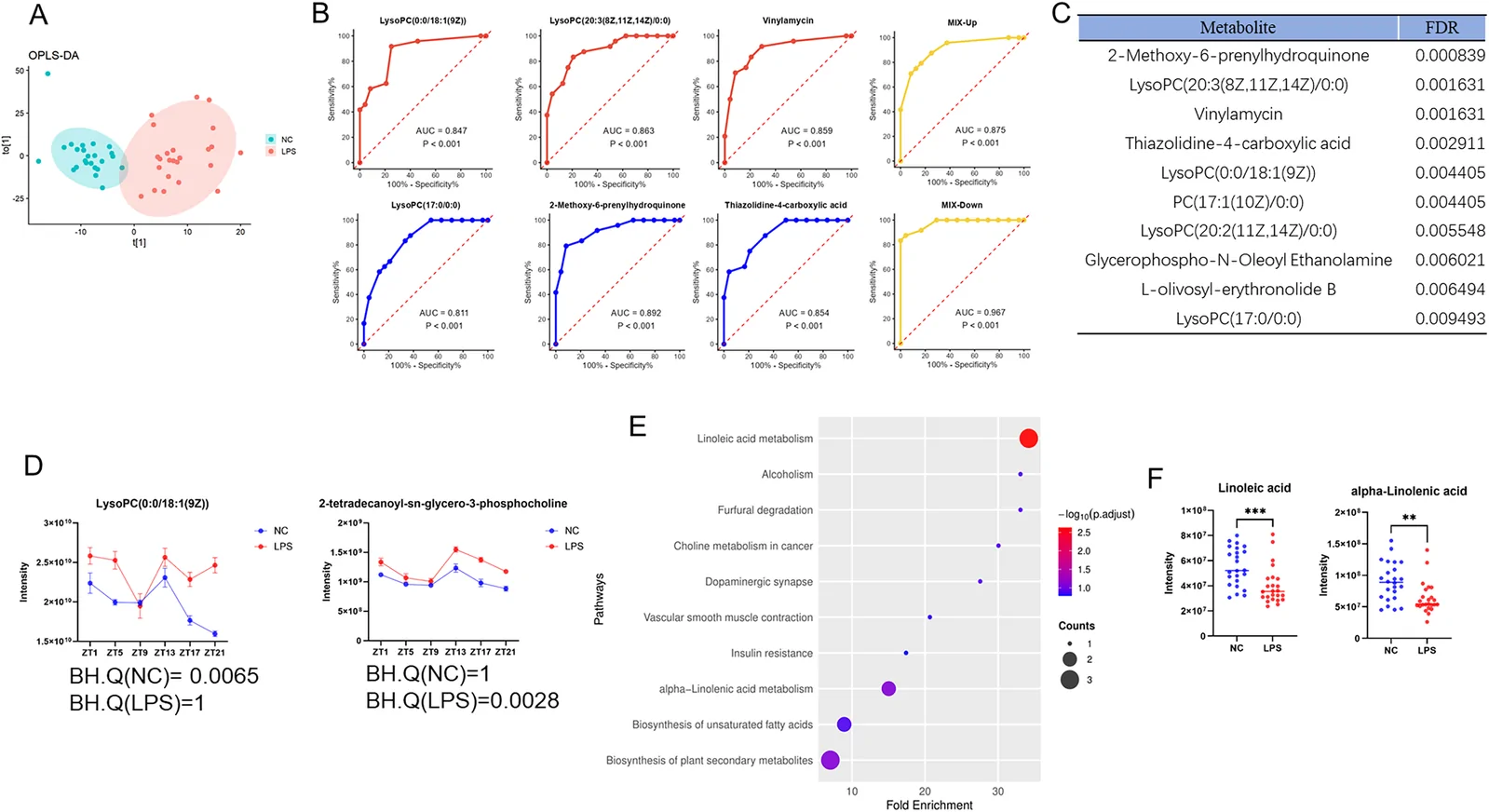
Plasma metabolomic analysis of liver samples combined across six circadian time points within a 24-hour cycle. **(A)** Orthogonal partial least squares discriminant analysis (OPLS-DA) score plot (n = 24): R²Y = 0.852, pR²Y = 0.010, Q² = 0.558, and pQ² = 0.005. **(B)** ROC curve analysis of top differential metabolites. **(C)** Top 10 differential metabolites in all time points. **(D)** Rhythmic characteristics of metabolites with significant differences. **(E)** KEGG enrichment of differential metabolites. **(F)** The level of Linoleic acid and alpha- Linolenic acid (n = 24). **p < 0.01; ***p < 0.001.

The rhythm analysis of the metabolome showed that there were 709 rhythm metabolites in the NC group and 835 in the LPS group, with a total of 391 rhythm metabolites in both groups (**Figure 6A**). Metabolome and transcriptome correlation analysis showed that the DEGs enrichment results of ZT21 were closer to the differential metabolites of ZT1, rather than ZT21. This may be due to the production of metabolites lagging behind changes in the transcriptome. At the same time, the circular plot showed that the differential metabolites of ZT1 were the most abundant at various time points (although not as prominent as those in the transcriptome), so we focused on the metabolomic results of ZT1 (**Figures 6B, C**). The ZT21 transcriptomic and ZT21 metabolomic datasets shared only two KEGG Level 2 categories, resulting in limited pathway-level concordance. Moreover, these shared pathways did not provide a coherent explanation for the transcriptome–metabolome relationship. In contrast, comparison with the ZT1 metabolome showed three shared KEGG Level2 categories and nine transcriptome-metabolome pathway connections, compared with four connections for the ZT21 comparison. We speculate that this may be due to the production of metabolites lagging behind changes in the transcriptome. Considering both the greater number of differential metabolites at ZT1 and the stronger pathway-level concordance with the ZT21 transcriptome, we focused on the ZT1 metabolomic results in the subsequent analyses (**Figures 6B,C**). Similar to the transcriptome findings, the heatmap of ZT1 metabolites in the NC group showed a significant loss of rhythmicity after LPS injection (**Figure 6D**). The clear separation of the NC and LPS groups in the PCA plot indicates distinct metabolic profiles between the two groups (**Supplementary Figure S8A**), and Lipid metabolism constituted the largest category of differential metabolites (**Supplementary Figure S8B**).

**Figure 6:**
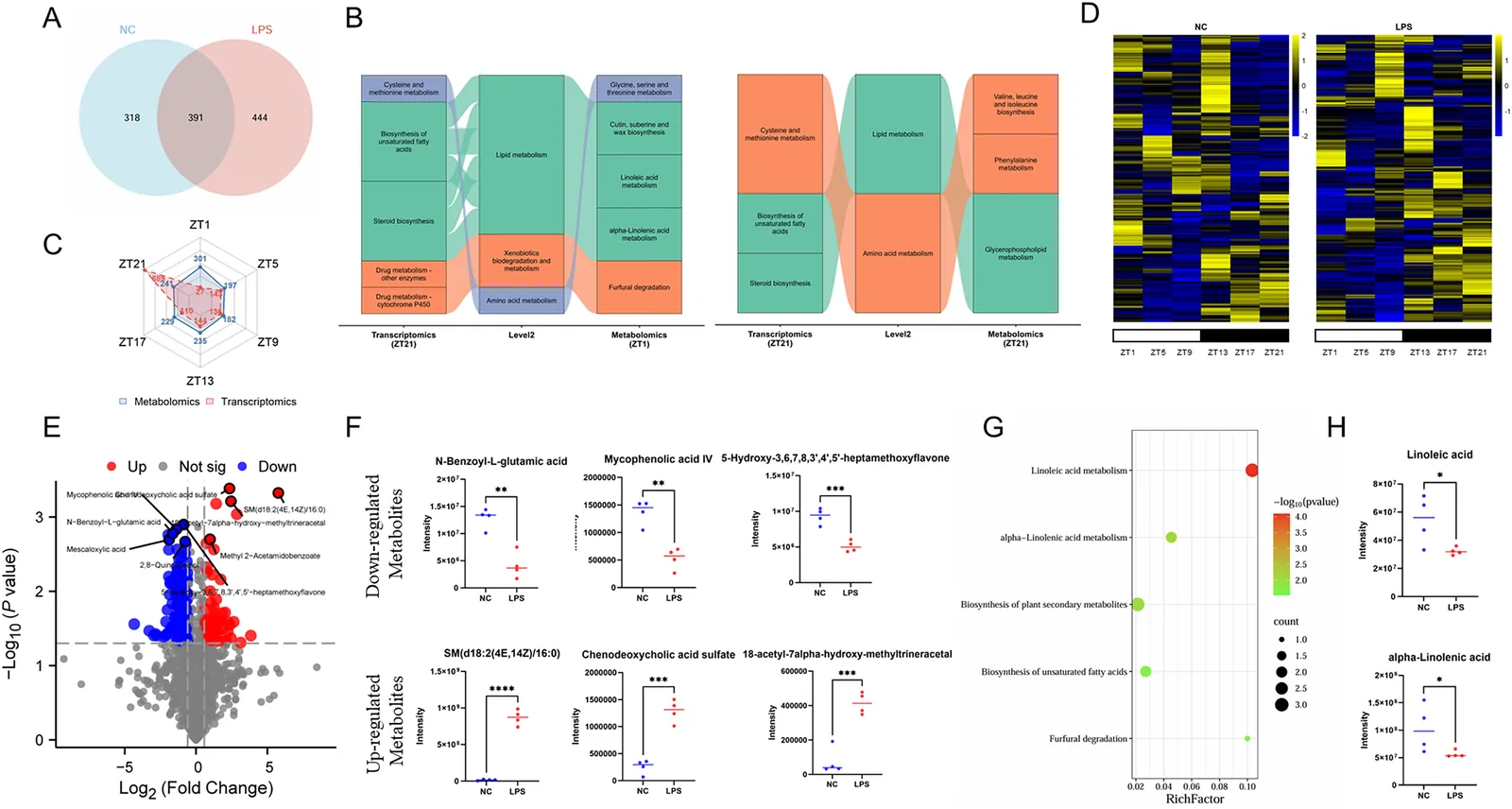
Plasma metabolomic rhythmicity across a 24-hour cycle and differences at ZT1. **(A)** Venn diagram of rhythmic metabolites in NC vs. LPS groups. **(B)** Sankey plots of pathway-level overlap between ZT21 liver transcriptomic KEGG enrichment (left) and plasma metabolomic KEGG enrichment (right) at ZT1 (left plot) and ZT21 (right plot). The middle column shows shared KEGG Level 2 categories. **(C)** Circular plot of DEGs and differential metabolites distribution. **(D)** Heatmap of topic rhythmic metabolites at ZT1 in NC and LPS groups. **(E)** Volcano plot of ZT1 differential metabolites between NC and LPS groups. **(F)** Intensities of top 3 upregulated and downregulated metabolites at ZT0 (n = 4). **(G)** KEGG pathway enrichment of ZT1 metabolites. **(H)** The level of Linoleic acid and alpha- Linolenic acid at ZT1 (n = 4). *p < 0.05; **p < 0.01; ***p<0.001; ****p < 0.0001.

The differential metabolites at different time points were also clustered in the heatmap (**Supplementary Figure S8C**), and the top 10 were displayed in the chord plot (**Supplementary Figure S8D**). N-Benzoyl-L-glutamic acid, Mycophenolic acid IV, and 5-Hydroxy-3,6,7,8,3’,4’,5’-heptamethoxyflavone were the most significantly downregulated metabolites, and SM (d18:2 (4E, 14Z) 16:0), and Chenodeoxycholic acid sulfate and 18-acetyl-7alpha-hydroxy-methyltrineracetal were most significantly up regulated (**Figures 6E,F**). KEGG pathway enrichment analysis showed that Linoleic acid metabolism remained the most enriched pathway, and Linoleic acid and alpha Linolenic acid remained the mosts significantly altered metabolites in these pathways (**Figures 6G, H**).

### 3.9 Transcriptome-metabolome integration analysis

Finally, we conducted the integrated analysis of the transcriptome and metabolome data at various time points and found significant enrichment of biosynthesis of the unsaturated fatty acid pathways (**Figure 7A**). This pathway involves *Acnat2* and *Acot3*, as well as the metabolites Linoleic acid and alpha Linolenic acid. Correlation analysis showed a strong correlation between Linoleic acid and alpha Linolenic acid and *Acnat2*, *Acot3* (**Figures 7B, C**), while ROC curve analysis confirmed that the use of Linoleic acid or alpha Linolenic acid alone can effectively distinguish each group (**Figure 7D**).

**Figure 7:**
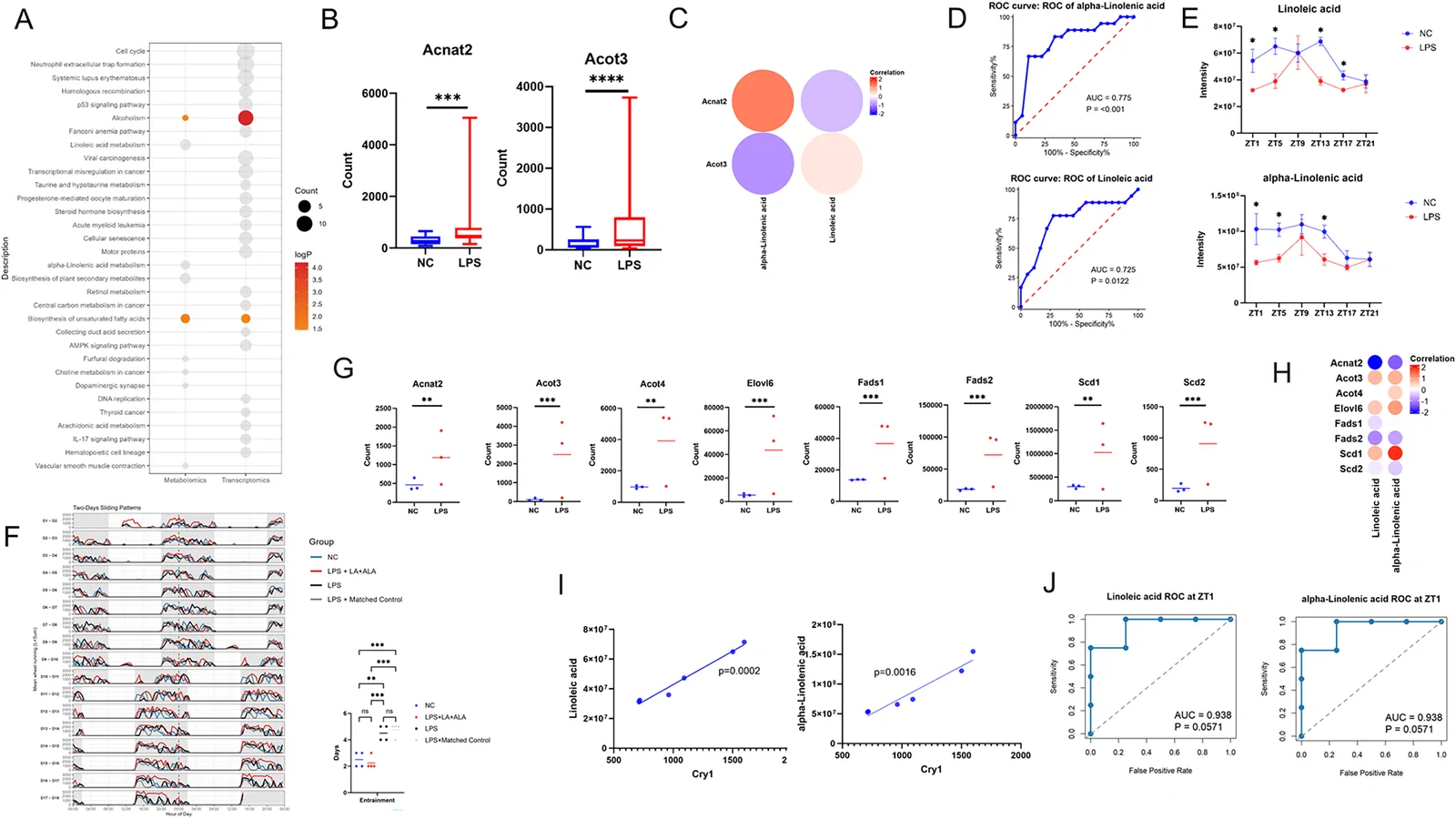
Transcriptome-Metabolome integration analysis. **(A)** Genes (n = 18) and metabolites (n = 24) co-enriched in the pooled analysis of six time points. **(B)** The level of Acnat2 and Acot3 in the pooled analysis of six time points (n = 18). **(C)** Correlation matrix of co-enriched genes (n = 18) and metabolites (n = 24). **(D)** ROC curve of Linoleic acid and alpha- Linolenic acid (n = 24) in the pooled analysis of six time points. **(E)** Linoleic acid and alpha- Linolenic acid intensity across all time points (n = 4). **(F)** Representative actograms showing activity rhythms in the NC, LPS, LPS + LA/ALA, and LPS + isocaloric matched-control groups following the phase shift. (n=4) **(G)** The level of DEGs in biosynthesis of unsaturated fatty acid pathway in ZT21 (n = 3). **(H)** Correlation matrix of co-enriched genes (ZT21, n=3) and metabolites (ZT1, n=4). **(I)** Correlation between Cry1 and Linoleic acid and alpha- Linolenic acid (n = 6). **(J)** ROC curves of Linoleic acid and alpha- Linolenic acid in ZT 1 (n = 4). ns p < 0.1; *p < 0.05; **p < 0.01; ***p < 0.001.

Given that the levels of Linoleic acid and alpha Linolenic acid constantly decrease at multiple time points 7 days after LPS injection (**Figure 7E**), we propose that plasma linoleic acid and alpha-linolenic acid represent sampling-time-independent candidate biomarkers associated with post-LPS residual circadian dysregulation. Therefore, we fed mice a diet supplemented with linoleic acid (LA) and α-linolenic acid (ALA) at a 1:1 ratio, based on their comparable reduction rates, after LPS administration. Compared with both the NC, LPS and LPS + isocaloric matched-control groups. LA+ALA supplementation significantly shortened the re-entrainment period, supporting the potential role of these fatty acids in promoting circadian adaptation. (**Figure 7F**).

When focusing specifically on ZT21, corresponding DEGs, including *Acnat2*, *Acot3*, and *Acot4*, etc. were selected based on the association analysis results in **Figure 6B** (**Figure 7G**). Correlation analysis showed that these genes were significantly correlated with Linoleic acid and alpha Linolenic acid (**Figure 7H**). It is worth noting that Linoleic acid and alpha Linolenic acid are also negatively correlated with *Cry1* (**Figure 7I**). ROC curve analysis further emphasized the enormous potential of these two metabolites as phase shift indicators (**Figure 7J**).

## 4 Discussion

Our research results showed that although acute inflammation, liver function, inflammatory factors, and melatonin levels returned to normal 7 days after LPS injection, the rhythm resynchronization time of the mice was significantly prolonged, indicating that persistent circadian dysfunction is not associated with acute organ damage. At the same time, plasma glucocorticoid levels continue to decrease, which may be due to acute glucocorticoid depletion and result in sustained clock asynchrony. It is worth noting that the general metabolic parameters and food intake among the groups were comparable, which confirms that the circadian rhythm disorder after sepsis is not caused by global metabolic failure, but rather by specific molecular rhythm remodeling and targeted metabolic reprogramming.

Transcriptomic analysis revealed that sepsis profoundly reshaped hepatic circadian homeostasis. The total number of rhythmic genes was markedly lower in the LPS group than in the NC group, accompanied by sustained low-grade inflammation, compensatory hepatocyte proliferation, and suppressed complement/coagulation pathways. Our dryR five-model stratification of hepatic rhythmic transcripts dissects distinct modes of sepsis-triggered circadian transcriptional remodeling based on binary rhythmic presence, oscillation amplitude and expression phase, resolving heterogeneous clock dysfunction undetectable by conventional Venn analyses.

The results of rhythm pattern classification further showed loss of rhythmicity in immune and cell-adhesion pathways, gain of rhythmicity in DNA replication and regeneration pathways and changes in amplitude or phase of cholesterol and amino acid related pathways, indicating that sepsis reconfigures the rhythmic landscape to support tissue repair while sacrificing normal circadian regulation. Transcriptomic analysis revealed that sepsis profoundly reshaped hepatic circadian homeostasis. The total number of rhythmic genes was markedly lower in the LPS group than in the NC group, accompanied by sustained low-grade inflammation, compensatory hepatocyte proliferation, and suppressed complement/coagulation pathways. Our dryR five-model stratification of hepatic rhythmic transcripts dissects distinct modes of sepsis-triggered circadian transcriptional remodeling based on binary rhythmic presence, oscillation amplitude and expression phase, resolving heterogeneous clock dysfunction undetectable by conventional Venn analyses. The results of rhythm pattern classification further showed loss of rhythmicity in immune and cell-adhesion pathways (Model 2), gain of rhythmicity in DNA replication and regeneration pathways (Model 3) and changes in amplitude or phase of cholesterol and amino acid related pathways (Model 5), indicating that sepsis reconfigures the rhythmic landscape to support tissue repair while sacrificing normal circadian regulation. In addition, Model 1 and Model 4 act as internal analytical controls, separating constitutively non-clock-coupled transcripts from unperturbed core clock housekeeping genes. dryR five-model classification moves beyond simple quantification of rhythmic gene loss to delineate three separable, functionally consequential modes of circadian transcriptome remodeling post-sepsis: immune rhythm ablation, regenerative pathway clock hijacking, and partial metabolic rhythm rewiring. Importantly, core clock genes maintained overall rhythmicity but exhibited marked time-specific dysregulation, most prominently at ZT21. The expression of *Bmal1*, *Clock*, *Per1*, and *Cry1* was significantly altered at ZT21 and showed strong discriminatory power between groups. Dynamic monitoring further revealed that *Bmal1*, *Cry1*, *Cry2*, and *Nr1d1* remained abnormally expressed for at least 11 days, suggesting that they are sensitive indicators of persistent circadian injury. Motif enrichment analysis identified *Cry1* as the top-predicted upstream regulator of ZT21-specific transcriptomic alterations. Genetic analysis using DKO mice offered partial experimental support, as only *Asap2* exhibited a significant genotype-treatment interaction. These results collectively suggest that *Cry1* may contribute to reshaping hepatic transcription at ZT21, yet additional non-*Cry1*-dependent pathways are also likely involved in driving post-sepsis hepatic transcriptome remodeling, which also reflects the complexity of rhythm regulation.

The gender differences in circadian rhythm regulation have received widespread attention. This study found that female mice showed better behavioral adaptation to changes in jet lag compared to male mice 7 days after the injection of LPS, which is consistent with previous research that females have stronger adaptability to jet lag (32). This gender characteristic may stem from differences in hormone regulation or gender specific transcriptional responses that challenge circadian rhythms, as estrogen has been shown to regulate clock gene expression in peripheral tissues (38). This finding indicates that the regulatory mechanism behind the phase shift is complex, and that there is a delay between behavior and molecular synchronization, which may be caused by intercellular coupling, thereby affecting the synchronization of the circadian clock (39). However, at the molecular level, the expression of core clock genes in female mice was similar to the results observed in male mice. These findings indicate that although female mice have better behavioral compensation ability, they also have incomplete molecular recovery after sepsis, and the molecular mechanism of circadian rhythm disorders is not affected by gender. However, lacking rigorous sex-treatment interaction statistical analysis, we cannot confirm that the *Cry1*-fatty acid regulatory axis and related biomarker characteristics are completely sex-independent. The clinical translational potential for female sepsis survivors requires further targeted verification.

This study also found that plasma LA and ALA level were biomarkers for circadian rhythm dysregulation after sepsis. ROC curve analysis confirmed that the use of LA and ALA alone or in combination showed strong diagnostic efficacy in distinguishing sepsis recovered mice from the control group. Unlike rhythmic transcripts or time-dependent hormones, LA and ALA reflect stable metabolic signatures and can be measured reliably at any time point, greatly facilitating clinical translation. These two essential polyunsaturated fatty acids are crucial for maintaining cell membrane fluidity, regulating inflammatory homeostasis, and serving as non-light circadian signals by modulating PER2 phosphorylation and core clock transcription (21–27). The sustained reduction of these genes after sepsis directly links metabolic reprogramming to dysfunction of the circadian rhythm clock and explains the long-term damage to rhythm synchronization. Most notably, in the mice recovered from sepsis, the LA and ALA concentrations continued to decrease at all the sampling time points except for ZT9 and ZT21, which is consistent with the findings of previous studies showing a decrease in plasma LA and ALA levels after sepsis (40). Meanwhile, ZT9 and ZT21, as representative time points for the late stages of light and darkness, indirectly reflect the reduced adaptability of the body to day night changes during the sepsis recovery period.

Furthermore, exogenous supplementation of LA and ALA at a 1:1 ratio significantly shortened the re-entrainment period, providing direct functional evidence that these metabolites are not merely biomarkers but also effective therapeutic agents for post-sepsis circadian disorders. This dual role, as a diagnostic marker and therapeutic target, highlights their unique translational value. The integration of transcriptomics and metabolomics shows that LA and ALA are closely related to *Acnat2* and *Acot3*, key enzymes in fatty acid metabolism, and are negatively correlated with *Cry1*. These results suggest a potential mechanism: Dysregulated *Cry1* may contribute to altered hepatic fatty-acid gene expression and subsequent LA/ALA reduction, ultimately impairing circadian rhythm synchronization. However, our DKO genetic experiments did not validate a universal *Cry1*-dependent regulation for these downstream molecular changes. Most tested target genes displayed non-significant genotype-treatment interactions, implying that *Cry1*-independent pathways also substantially mediate post-sepsis hepatic transcriptome and metabolic reprogramming. Hence, this *Cry1*-essential fatty-acid axis remains a correlative-derived hypothesis that needs further dedicated molecular and genetic validation.

It is worth noting that although plasma TNF-α and IL-6 returned to baseline levels seven days after LPS injection, hepatic transcriptomics still showed enrichment of inflammatory pathways, indicating persistent tissue-level low-grade hepatic inflammation even after resolution of systemic acute inflammation. Therefore, reduced circulating LA and ALA may not exclusively report circadian dysfunction; they could also be influenced by residual tissue inflammation or broader systemic metabolic reprogramming. Ideally, within-animal correlation between individual plasma LA/ALA concentrations and circadian re-entrainment capacity would strengthen evidence for biomarker specificity. However, our metabolomics requires terminal euthanasia for plasma collection. This terminal sampling design prevents us from measuring plasma fatty-acid concentrations and subsequent behavioral re-entrainment within the same animal, precluding this paired correlation analysis. While our dietary supplementation experiment supports a functional link between these essential fatty acids and circadian re-entrainment, we cannot formally rule out contributions from residual inflammation when interpreting LA/ALA as biomarkers of circadian impairment. Future studies with serial non-terminal blood sampling will help further resolve the specificity of LA/ALA for circadian versus inflammatory or metabolic perturbations in sepsis survivors.

This study utilized non-lethal LPS-induced endotoxemia, a well-established experimental surrogate for systemic inflammatory states, rather than a live-pathogen-driven infection model of true sepsis. Of note, the widely-used cecal ligation and puncture polymicrobial sepsis model frequently produces high mortality rates, and it is technically challenging to generate stable non-lethal CLP conditions suitable for investigating long-term post-recovery sequelae. This practical constraint partially motivated our choice of non-lethal LPS administration, which enables consistent animal survival and permits assessment of phenotypes during the recovery phase. Nevertheless, LPS endotoxemia triggers acute systemic inflammation via toll-like receptor-4 activation, yet it lacks key features of clinical sepsis including live microbial burden, pathogen clearance processes, tissue bacterial invasion, dynamic host-pathogen interactions, and antibiotic-treatment-related physiological perturbations (41–43). Future work will focus on developing refined live-pathogen-driven non-lethal sepsis models. Additional validation using such improved infection models will be critical to further verify whether the *Cry1*-essential fatty-acid regulatory axis and plasma LA/ALA biomarkers are generalizable across diverse pre-clinical sepsis paradigms before clinical translation to human sepsis survivors.

This study systematically explored the residual characteristics of circadian rhythm regulation in male mice after sepsis recovery through multi-omics analysis, and found that ZT21 is a key time window for detecting circadian rhythm disorders, and that *Cry1* may be the core regulatory gene involved in this process. LA and ALA can be used as sensitive biomarkers for post-sepsis circadian rhythm disorders, and exogenous supplementation can effectively improve the rhythm synchronization ability of sepsis survivors. Female mice exhibit partial molecular consistency but distinct behavioral performance in circadian recovery. Further studies with designed sex-treatment interaction assays are needed to determine the sex universality of the above regulatory mechanism and intervention strategy. Our multi-omics analyses uncover correlative evidence supporting a putative *Cry1*-essential fatty-acid axis in post-sepsis circadian impairment, and identify plasma LA and ALA as promising sampling-time-independent biomarkers. Exogenous LA+ALA supplementation rescued circadian re-entrainment defects in sepsis recovered mice. Further work is required to dissect causal contributions of *Cry1*-dependent versus *Cry1*-independent pathways to these metabolic and circadian phenotypes. In the future, we will further explore the specific molecular mechanism of LA, ALA and *Cry1* in regulating post-sepsis circadian rhythm, and verify the clinical application value of these findings in sepsis survivors, so as to improve the long-term recovery of sepsis patients.

## Data Availability

The datasets presented in this study can be found in online repositories. The names of the repository/repositories and accession number(s) can be found in the article/**Supplementary Material**.
