# Supplementary material for: Integrative transcriptomics and metabolomics reveal Cry1-associated hepatic alterations and plasma essential fatty acids as biomarkers of residual circadian disruption post-sepsis
Source: Front Endocrinol (Lausanne). 2026 Oct 1;17:1916119. doi: 10.3389/fendo.2026.1916119 (PMC13668567; doi:10.3389/fendo.2026.1916119)
Supplement: Supplementary file 1 [file Supplementaryfile1.docx]

Supplementary Material

This file includes:

Supplementary Table 1

Supplementary Figure 1

Supplementary Figure 2

Supplementary Figure 3

Supplementary Figure 4

Supplementary Figure 5

Supplementary Figure 6

Supplementary Figure 7

Supplementary Figure 8

**Supplementary Table1: Primers**

| **Species** | **The primer (5’-3’)** | **Sense** | **Anti-sense** | **Company and location** |
| --- | --- | --- | --- | --- |
| Mouse | Bmal1 | ACGACATAGGACACCTCGCAGA | TCCTTGGTCCACGGGTTCA | TaKaRa, CN |
| Mouse | Clock | AGCTGCATATTGCCGTTGTAATTG | CTGAGTGAAGGCATGCTGGTG | TaKaRa, CN |
| Mouse | Cry1 | GAGGACAGATCCCAATGGAGACTA | TGGTGCATTCCAAGGATCGTA | TaKaRa, CN |
| Mouse | Cry2 | TTCTGCGTCCAGGATTTAGTTTG | GGAATGGGCCAGCTTGTCA | TaKaRa, CN |
| Mouse | Per1 | GTGAGGAGACTGCTGAGCCAGA | AGGTCGCTGGAGCCTGAAAGT | TaKaRa, CN |
| Mouse | Nr1d1 | TCGTTAAGGCTGGCACCTTTG | GTAGGTTGTGCGGCTCAGGAA | TaKaRa, CN |
| Mouse | Nr1d2 | TGCTACAGGATTTGAACTTCTGTGG | CACTGAACGTAAGCAGCAGTTATGG | TaKaRa, CN |
| Mouse | Dbp | AAGCATTCCAGGCCATGAGAC | TTCTTGTACCTCCGGCTCCAG | TaKaRa, CN |
| Mouse | Ager | AGGAGGTCAAGTCCAACTACC | TAGCTTCCCTCAGACACACAT | TaKaRa, CN |
| Mouse | Src | CTAGACGGGAATCAGAGCGG | TGGCATTGTCGAAGTCGGAT | TaKaRa, CN |
| Mouse | Gsdmd | CCCGTTATTCATGTGTCAACCTGT | GCCCTGAATGTTCCCATCGAC | TaKaRa, CN |
| Mouse | Pik3r5 | GCAGAGAGATGCTCACCTTCA | TACCTCTACCGGGTTCAGCA | TaKaRa, CN |
| Mouse | Asap2 | CACGGCCTCTAGCTTCACCA | AGAACCATCCGGTCCACGTC | TaKaRa, CN |
| Mouse | Tymp | GCACGCGGTGATAGATGGA | GCACAGATGTCTCTTCTAGGTTCA | TaKaRa, CN |
| Mouse | Mafb | CACCAAGGACGAGGTGATCC | GTGATGTTTCTGCTGGACGC | TaKaRa, CN |
| Mouse | Clpx | GTCTTGTTTGTGGCATCTGGT | ATGGTGTTCCAAAGCCGAGA | TaKaRa, CN |
| Mouse | β-actin | CATCCGTAAAGACCTCTATGCCAAC | ATGGAGCCACCGATCCACA | TaKaRa, CN |
|  |  |  |  |  |

**Figure S1. Organ and metabolic changes after LPS injection for 7 days.** (A) Schematic diagram of the experiment. (B) Spleen and liver weight ratios (n=24). (C) Plasma melatonin levels (n=4). (D) Plasma corticosterone levels (n=4). (E) Plasma ALT, AST levels (n=9). (F) Plasma TNFα, IL-6 levels (n=9). (G) Respiratory exchange ratio (n=3). (H) VCO₂ production (n=3). (I) food intake (n=3).

**Figure S2. Behavioral changes after LPS injection for 7 days.** (A-B) Actograms and activity quantification (n=3).

**Figure S3. OPLS-DA and DEG analysis of liver samples combined across six circadian time points within a 24-hour cycle.** (A)PCA plot of transcriptomic profiles from the NC and LPS groups across all circadian time points (n = 18). (B) Orthogonal partial least squares discriminant analysis (OPLS-DA) score plot of transcriptomic profiles from the NC and LPS groups across all circadian time points (n = 18): R²Y = 0.968, pR²Y = 0.025, Q² = 0.497and pQ² = 0.005. (C) Top 10 DEGs.

**Figure S4. DryR modeling of liver transcriptome across a 24-hour cycle.** (A) Gene counts in dryR models. (B-C) Heatmap and KEGG enrichment of Model 1. (D-F) Heatmap, phase plot, and KEGG enrichment of Model 4. (G-I) Model 5: heatmap, phase plot, and pathway enrichment of Model 5. (n=3).

**Figure S5. Core clock gene rhythmicity and phase in the liver transcriptomes across a 24-hour cycle.** (A) Expression levels of core clock genes in transcriptome (n=3). B) Peak phases of core clock genes from dryR analysis. * P <0.05; ** P <0.01.

**Figure S6. Responses of female mice to jet lag.** (A-B) Actograms and activity before/after shift (n=3). (C-E) RER ; VCO₂ and Feeding. (F) qPCR analysis of core clock genes 7 days after LPS injection (n = 3).. * P< 0.05; ** P< 0.01.

**Figure S7. Plasma metabolomic analysis of liver samples combined across six circadian time points within a 24-hour cycle.** (A)PCA plot across all circadian time points (n = 24). (B) Volcano plot of differential metabolites (n=24). (C) Level of top 3 up/downregulated metabolites (n=24). (D) Heatmap of rhythmic profiles of differential metabolites. (E) Classification of differential metabolites. (F) Linoleic acid metabolism pathway metabolites (n=24). * P <0.05; ** P <0.01; *** P <0.001.

**Figure S8. Plasma metabolomics at ZT1.** (A) PCA of metabolitesat ZT1 (n=4). (B) Classification of ZT1 differential metabolites. (C) Heatmap of top 50 ZT1 metabolites. (D) Chord diagram of top 20 ZT1 metabolites.
